# Periodontitis may predict the use of prescription medicines later in life, a database study

**DOI:** 10.3389/fphar.2023.1146475

**Published:** 2023-03-13

**Authors:** Freja Frankenhaeuser, Birgitta Söder, Håkan Källmén, Esa R. Korpi, Jukka H. Meurman

**Affiliations:** ^1^ Department of Oral and Maxillofacial Diseases, University of Helsinki and Helsinki University Hospital, Helsinki, Finland; ^2^ Department of Dental Medicine, Karolinska Institutet, Stockholm, Sweden; ^3^ Stockholm Region Health Services, Stockholm, Sweden; ^4^ Department of Pharmacology, University of Helsinki, Helsinki, Finland

**Keywords:** periodontitis, medicines, systemic disease, oral health, prescription of drugs

## Abstract

Medications used for the treatment of diseases also affect oral health. We investigated how having/not having periodontitis at baseline in 1985 was associated with purchases of medicines in the long term. The study paradigm is in the oral health-systemic health connections. We hypothesized that periodontitis links to purchases of medicines later in life. The study cohort consisted of 3,276 individuals from the greater Stockholm area, Sweden. Of them, 1,655 were clinically examined at baseline. Patients were followed-up for >35 years, using the national population and patient registers. The burden of systemic diseases and purchases of medicines were statistically analyzed comparing patients with (*n* = 285) and without (*n* = 1,370) periodontitis. The results showed that patients with periodontitis had purchased more of certain medications than non-periodontitis patients. Periodontitis patients purchased significantly more drugs used in diabetes (*p* = 0.035), calcium channel blockers (*p* = 0.016), drugs acting on the renin-angiotensin system (*p* = 0.024), and nervous system drugs (*p* = 0.001). Hence, patients with periodontitis indeed had purchased specific medications statistically significantly more than the periodontally healthy ones. This indicates that periodontitis, over time, might increase the risk for systemic diseases with the subsequent need for medication.

## 1 Introduction

Periodontitis or periodontal disease is a chronic inflammation of the gums and tooth supporting tissues, leading to attachment and bone loss, due to the immune response caused by accumulations of bacterial biofilm on the teeth. Numerous studies have verified the link between poor oral health and systemic health (Meurman and Bascones-Martinez, 2021). In particular, periodontal disease is associated with cardiovascular diseases and diabetes, but also with many other diseases (Humphrey et al., 2008; Pussinen et al., 2022). Recently, periodontitis was shown to associate even with the outcome of COVID-19 (Marouf et al., 2021; Gupta et al., 2022). For example, in the study of Orilisi and coworkers (Orilisi et al., 2021) it was shown that patients with oral health problems were referred to intensive care more often than those without. The pathomechanism here involved is the chronic oral infection that upregulates many cytokines and inflammatory mediators with subsequent systemic organ effects (Hansen and Holmstrup, 2022).

Little research is aimed at periodontitis and its effect on medication use (Wang et al., 2020). Anticholinergic and psychiatric medications are the most discussed in this context. Well-known oral side effects of drugs, in general, are hyposalivation, xerostomia, gingival overgrowth, hypersalivation, lichenoid reactions, and osteonecrosis of the jaws (Kaur et al., 2010; Miranda-Rius et al., 2015; Trackman and Kantarci, 2015; Glick et al., 2020; Yuan and Woo, 2020). Drugs for the treatment of hypertension and diabetes, are examples of medication that may cause hyposalivation with subsequent subjective xerostomia (Närhi et al., 1992). Low salivary flow presents a risk for dental diseases, in particular caries, but also periodontal health may worsen if the patient has dry mouth (Mizutani et al., 2015; Pająk-Łysek et al., 2021). However, it should be emphasized that the link between periodontitis and saliva secretion is not as straightforward as it is with caries (Rees, 1998).

In the present longitudinal cohort study, we investigated the association between baseline periodontal status and the purchase of prescription medicines later in life. We hypothesized that people with poor periodontal health would present with more systemic diseases and, consequently, would need medication more often than those who originally were periodontally healthy. The subjects of our study had been followed up for more than 35 years and the investigation was based on different national patient registers in Sweden.

## 2 Material and methods

### 2.1 Subjects of the cohort

**FIGURE 1:**
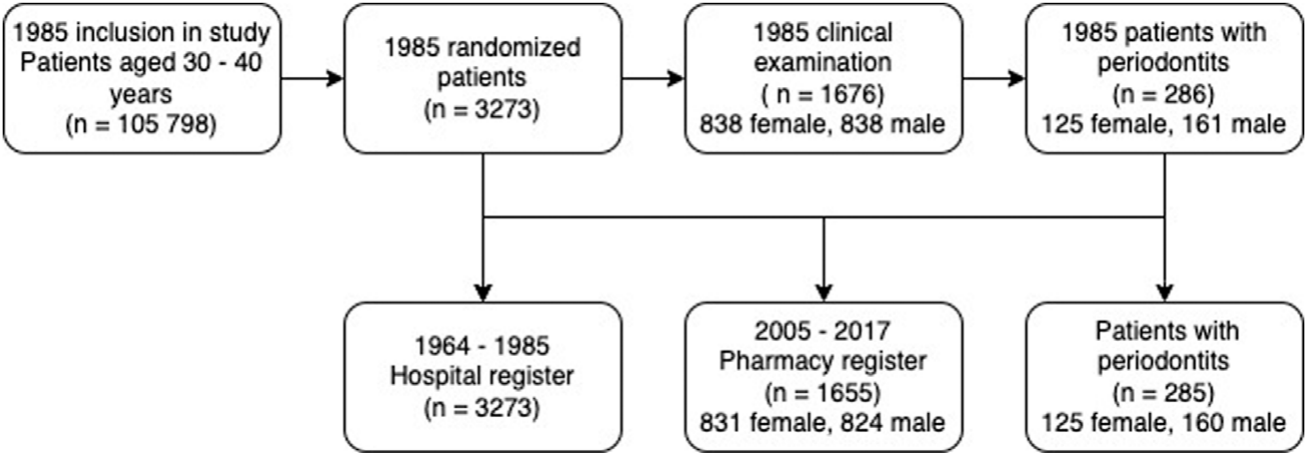
Flow chart of cohort and registers available. In the present study, we mainly focused on the pharmacy register.

Our database originates from the year 1985 and consists of 3,273 randomized subjects enrolled from the Stockholm metropolitan area, Sweden. All our participants were born from 1945–1954 on the 20th of each month. The basic cohort size was 105,798 persons (Söder et al., 1994). Since 1985 the subjects’ health parameters had been followed-up, now for over 35 years. In the present study, our sample consists of 1,655 patients, 824 men, and 831 women. Of these patients, 285 had had periodontitis at baseline in 1985 (Figure 1). In 1985, 1,676 patients were clinically examined at baseline. However, due to the fallout of 21 patients, our clinically examined and followed-up study group consists of 1,655 patients.

### 2.2 The drug (pharmacy) register

The database used for examining the prescription of medication among patients is the Swedish National Pharmacology register. This register consists of the 1,655 subjects’ total procurement history of medications during the timespan of the years 2005–2017. The register contains altogether 469,789 purchases with 975 individual Anatomical Therapeutic Chemical (ATC) codes. For analysis, procurement of medication or the medication class was coded as one and no procurement as 0.

### 2.3 Periodontitis diagnosis

In the clinical oral examinations in 1985, the patients underwent oral examination where plaque index, gingival index (GI), and periodontal pocket probing (CAL) were registered. Periodontal pockets (PD) over 5 mm were recorded. A dichotomized variable was created for statistical analysis, where patients with periodontitis were coded one and periodontally healthy control subjects 0.

### 2.4 Socio-economic status

In the baseline data from 1985, patients were divided into higher or lower socio-economic classes based on income and level of education. Patients with a lower level of education and low or no income were coded as having lower socio-economic status and ones with income and a higher level of education were coded as higher. This was used as a covariate in our research. One dichotomized variable was created from the original baseline variable to indicate the patients’ economic status so that the subjects with high socioeconomic status were coded 0 and those with low 1, respectively.

### 2.5 Diagnoses before 1985

To control for diseases and subsequent systemic medicine use before 1985, the Swedish National Hospital register was used. Patients with at least one diagnosis given in hospital care were categorized in a dichotomized variable: a hospital diagnosis before 1985 was coded as one and no hospital diagnosis as 0, respectively. Hospitalization due to poisoning or pregnancy was disregarded.

### 2.6 Tobacco products

At baseline, the patient´s use of tobacco products was registered. A dichotomized tobacco products variable was created where patients who were smoking or using Swedish snus in 1985 were coded as one and patients not using tobacco products were coded as 0.

### 2.7 Gender

Research has shown a difference in oral health among men and women, where men often have worse oral health. To take this into account a dichotomized variable was created where women were coded as 0 and men as 1.

### 2.8 Statistical analyses

Descriptive statistics, Chi^2^, p-tests, and logistic regression analyses were conducted in SPSS 28.0 software. A single-sided hypothesis was used in this study, resulting in the use of one-tailed tests. Descriptive statistics as frequencies were conducted, differences between groups were tested by Mann-Whitney U-tests, and differences in the distribution of data were analyzed by Chi^2^. Logistic regression analyses of procurement of medicines with periodontitis as explaining variable were controlled for gender (men 1, women 0), tobacco products (yes 1, no 0), socio-economic (lower 1, higher 0), and if the subject had a diagnosis of systemic disease before 1985 (yes 1, no 0). Data reorganization and summation of the different registers were made in Visual Studio Code 2, Python 3.9.10 64-bit.

## 3 Results

The number of patients with periodontitis, tobacco usage, gender, and diagnoses at baseline are given in Table 1. Patients with periodontitis had not purchased more medications than the non-periodontitis subjects between the years 2005–2017. Fewer patients with periodontitis had acquired medications in general than periodontally healthy individuals (89.5% vs 93.4%). The medication categories most patients had purchased during the timespan was ATC category J, anti-infectives for systemic use (n = 1,379), as can be seen in Table 2. The second in frequency was medicines used for diseases of the nervous system (n = 1,197) and, third, respiratory medications (n = 1,157). Comparing the purchases by periodontitis patients with those of the periodontally healthy, periodontitis patients had purchased more drugs of the ATC category C, cardiovascular system, L, antineoplastic and immunomodulating agents, and P, antiparasitic products, insecticides and repellents (Table 2).

**TABLE 1:** Basic characteristics of the subjects. The table presents patients that have purchased medicines between the years 2005–2017. Data are given as n (%). Only *p*-values in line with our hypothesis are presented, due to our single sided hypothesis.

	Total (n = 1,665)	No-periodontitis (n = 1,370)	Periodontitis (n = 285)	*p*-value
Female	831	706 (85.0%)	125 (15.4%)	
Male	824	664 (80.6%)	160 (19.4%)	0.009
Non-smoker	618	907 (86.4%)	143 (13.6%)	
Smoker	605	463 (76.5%)	142 (23.5%)	<0.001
No earlier diagnosis	1,070	884 (82.6%)	186 (17.4%)	
Diagnosis in 1985	585	486 (83.1%)	99 (16.9%)	
Have not purchased medication	120	90 (75.0%)	30 (25.0%)	
Have purchased medication(s)	1,535	1,280 (83.4%)	255 (16.6%)	
Higher socio-economic status	1,315	1,097 (83.4%)	273 (16.6%)	
Lower socio-economic status	340	218 (80.3%)	67 (19.7%)	0.087

**TABLE 2:** Distributions of different medication groups purchased in the ATC categories. Only *p*-values in line with our hypothesis are presented, due to our single sided hypothesis.

Alimentary tract and metabolism	Total	No periodontitis		Periodontits		*p*-value
Have not purchased	540	441	(81.7%)	99	(18.3%)	
Have purchased	1,115	929	(83.3%)	186	(16.7%)	
Blood and blood forming organs						
Have not purchased	889	739	(83.1%)	150	(16.9%)	
Have purchased	766	631	(82.4%)	135	(17.6%)	0.344
Cardiovascular system						
Have not purchased	640	539	(84.2%)	101	(15.8%)	
Have purchased	1,015	831	(81.9%)	184	(18.1%)	0.109
Dermatologicals						
Have not purchased	683	566	(82.9%)	127	(18.6%)	
Have purchased	962	804	(83.6%)	158	(16.4%)	
Genito-urinary system and sex hormones						
Have not purchased	835	689	(82.5%)	146	(17.5%)	
Have purchased	820	681	(83.0%)	139	(17.0%)	
Systemic hormonal preparations, excluding sex hormones and insulins						
Have not purchased	1,078	878	(81.4%)	200	(18.6%)	
Have purchased	557	492	(88.3%)	85	(15.3%)	
Anti-infectives for systemic use						
Have not purchased	276	228	(82.6%)	48	(17.4%)	
Have purchased	1,379	1,142	(82.8%)	237	(17.2%)	
Antineoplastic and immunomodulating agents						
Have not purchased	1,481	1,231	(83.1%)	250	(16.9%)	
Have purchased	174	139	(79.9%)	35	(20.1%)	0.143
Musculo-skeletal system						
Have not purchased	502	415	(82.7%)	87	(17.3%)	
Have purchased	1,153	955	(82.8%)	198	(17.2%)	
Nervous system						
Have not purchased	458	377	(82.3%)	81	(17.7%)	
Have purchased	1,197	993	(83.0%)	204	(17.0%)	
Antiparasitic products, insecticides and repellents						
Have not purchased	1,301	1,084	(83.3%)	217	(16.7%)	
Have purchased	354	286	(80.8%)	68	(19.2%)	0.132
Respiratory system						
Have not purchased	498	404	(81.1%)	94	(18.9%)	
Have purchased	1,157	966	(83.5%)	191	(16.5%)	
Sensory organs						
Have not purchased	813	668	(82.2%)	145	(17.8%)	
Have purchased	842	702	(83.4%)	140	(16.6%)	
Various						
Have not purchased	1,633	1,350	(82.7%)	283	(17.3%)	
Have purchased	22	20	(90.9%)	2	(9.1%)	

Looking at the pharmaceutical groups of drugs according to the ATC classification, there were medicines in five different groups that had been purchased more by the periodontally diseased patients. These were drugs used for diabetes (*p* = 0.035), calcium channel blockers (*p* = 0.016), drugs acting on the renin-angiotensin system (*p* = 0.032), lipid modifying agents (*p* = 0.024), and drugs used for other nervous system diseases (*p* = 0.001). Another fourteen different categories of medications used more by the periodontitis patients (*p* = 0.102–0.462) were cardiovascular drugs and beta-blocking agents, in particular, antineoplastic agents, drugs for endocrine diseases, immunosuppressants, anti-inflammatory, and antirheumatic drugs, topical products for joint and muscular pain, muscle relaxants, antigout preparations, analgesics, antiepileptics, antiparkinson drugs, psycholeptics (antipsychotics, anxiolytics, hypnotics, and sedatives) and antiprotozoals. The details age given in Table 3.

**TABLE 3:** Drugs most frequently purchased within the ATC categories. The statistically significant distribution in favor of periodontitis patients are bolded.

ATC classification	Total	Non-periodontitis		Periodontitis		*p*-value
Alimentary tract and metabolism						
Drugs used in diabetes						
Have not purchased	1,499	1,249	(83.3%)	250	(16.7%)	
Have purchased	156	121	(77.6%)	35	(22.4%)	0.035
Cardiovascular system						
Cardiac therapy						
Have not purchased	1,444	1,198	(83.0%)	246	(17.0%)	
Have purchased	211	172	(81.5%)	39	(18.5%)	0.300
Beta blocking agents						
Have not purchased	1,178	982	(83.4%)	196	(16.6%)	
Have purchased	477	388	(81.3%)	89	(18.7%)	0.162
Calcium channel blockers						
Have not purchased	1,271	1,066	(83.9%)	205	(16.1%)	
Have purchased	384	304	(79.2%)	80	(20.8%)	0.016
Agents acting on the renin–angiotensin system						
Have not purchased	1,055	887	(84.1%)	168	(15.9%)	
Have purchased	600	483	(80.5%)	117	(19.5%)	0.032
Lipid modifying agents						
Have not purchased	1,139	957	(84.0%)	182	(16.0%)	
Have purchased	519	182	(35.1%)	103	(19.8%)	0.024
Antineoplastic and immunomodulating agents						
Antineoplastic agents						
Have not purchased	1,601	1,328	(82.9%)	273	(17.1%)	
Have purchased	54	42	(77.8%)	12	(22.2%)	0.161
Endocrine therapy						
Have not purchased	1,588	1,315	(82.8%)	273	(17.2%)	
Have purchased	67	55	(82.1%)	12	(17.9%)	0.440
Immunosuppressants						
Have not purchased	1,601	1,326	(82.8%)	275	(17.2%)	
Have purchased	54	44	(81.5%)	10	(18.5%)	0.399
Musculo-skeletal system						
Anti-inflammatory and antirheumatic products						
Have not purchased	561	467	(83.2%)	98	(17.5%)	
Have purchased	1,094	903	(82.5%)	191	(17.5%)	0.360
Topical products for joint and muscular pain						
Have not purchased	1,484	1,230	(82.9%)	254	(17.1%)	
Have purchased	171	140	(81.9%)	31	(18.1%)	0.370
Muscle relaxants						
Have not purchased	1,484	1,231	(83.0%)	253	(17.0%)	
Have purchased	171	139	(81.3%)	32	(18.7%)	0.293
Antigout preparations						
Have not purchased	1,587	1,316	(82.9%)	271	(17.1%)	
Have purchased	68	54	(79.4%)	14	(20.6%)	0.227
Nervous system						
Analgesics						
Have not purchased	643	538	(83.7%)	105	(16.3%)	
Have purchased	1,012	832	(82.2%)	180	(17.8%)	0.222
Antiepileptics						
Have not purchased	1,526	1,268	(83.1%)	258	(16.9%)	
Have purchased	129	102	(79.1%)	27	(20.9%)	0.123
Anti-parkinson drugs						
Have not purchased	1,607	1,331	(82.8%)	276	(17.2%)	
Have purchased	48	39	(81.3%)	9	(18.8%)	0.388
Psycholeptics						
Have not purchased	974	807	(82.9%)	167	(17.1%)	
Have purchased	681	563	(82.7%)	118	(17.3%)	0.462
Other nervous system drugs						
Have not purchased	1,548	1,293	(83.5%)	255	(16.5%)	
Have purchased	107	77	(72.0%)	30	(28.0%)	0.001
Antiparasitic products, insecticides and repellents						
Antiprotozoals						
Have not purchased	1,312	1,094	(83.4%)	218	(16.6%)	
Have purchased	343	276	(80.5%)	67	(19.5%)	0.102

When looking at the ATC subclasses and specific drug preparations, differences were also detected in the purchase numbers of medicines between the periodontitis and non-periodontitis groups. In addition to the results given in Table 3, purchases of 18 specific drug preparations were significantly more common among periodontitis patients. They purchased more of the following preparations: insulin (2.46% vs. 0.95%, *p* = 0.017), calcium channel blocker felodipine (8.42% vs. 5.62%, *p* = 0.036), angiotensin-converting enzyme (ACE) inhibitor ramipril (4.91% vs. 2.99%, *p* = 0,05), HMG CoA reductase inhibitor simvastatin (29.4% vs. 22.6%, *p* = 0.007), opioid analgesics ketobemidone (3.16% vs. 1.09%, *p* = 0.004) and fentanyl (1.75% vs. 0.58%, *p* = 0.021), nicotine dependence drug varenicline (5.26%–2.04%, *p* = 0.001), and the nitroimidazole antibiotic metronidazole (17.5% vs. 12.4%, *p* = 0.01). Eleven preparations were excluded from the list because less than five subjects had purchased them.

In total, 77 different individual preparations had been purchased more often by the periodontitis patients than the periodontally healthy. Among the 59 preparations that had not been significantly more purchased there were 17 different individual diabetes medications (*p* = 0.017–0.323), the channel blocker amlodipine (22.5% vs 18.8%, *p* = 0.076), ACE-inhibitor enalapril (25.6% vs 21.3%, *p* = 0.056), disulfiram (1.4% vs 0.7%, *p* = 0.097), analgesic morphine, and antispasmodics (2.5%–1.5%, *p* = 0.114).

Odds ratios for the different categories of medications mainly linked to periodontitis are given in Table 4 and Table 5. In the main categories and subtypes of medications purchased, positive odds ratios, with a confidence interval over one, were not detected. Regarding individual preparations, only simvastatin (ATC class C10AA01, OR = 1.4; CI = 1.04–1.86), ketobemidone (ATC class N02AB01, OR = 3.32; CI = 1.48–7.86) and metronidazole (ATC class P01AB01, OR = 1.46; CI = 1.03–2.08) showed positive odds ratios.

**TABLE 4:** Linear regressions and odds ratios (OR) with confidence intervals (CI) of drug categories associated with having periodontitis in 1985.

	Cofactors	OR	95% CI for OR	
Nervous system			Lower	Upper
	Periodontitis	0.957	0.714	1.28
	Male	0.553	0.442	0.692
	Smoker	1.49	1.17	1.89
	Prior diagnosis to 1985	1.54	1.21	1.96
	Lower socioeconomic	0.634	0.487	0.825
Alimentary tract and metabolism				
	Periodontitis	0.932	0.707	1.22
	Male	0.612	0.495	0.756
	Smoker	1.06	0.850	1.32
	Prior diagnosis to 1985	1.61	1.28	2.02
	Lower socioeconomic	0.653	0.507	0.840
Cardiovascular system				
	Periodontitis	1.16	0.888	1.52
	Male	0.989	0.808	1.20
	Smoker	1.19	0.969	1.47
	Prior diagnosis to 1985	1.44	1.16	1.78
	Lower socioeconomic	0.796	0.622	1.01
Antineoplastic and immunomodulating agents				
	Periodontitis	1.27	0.852	1.90
	Male	0.633	0.457	0.877
	Smoker	1.12	0.809	1.55
	Prior diagnosis to 1985	1.25	0.903	1.73
	Lower socioeconomic	0.814	0.538	1.22

**TABLE 5:** Linear regressions and odds ratios (OR) with confidence intervals (CI) of individual medications associated with having periodontitis in 1985.

	Cofactors	OR	95% CI for OR	
Simvastatin				
	Periodontitis	1.39	1.04	1.86
	Male	1.14	0.909	1.44
	Smoker	1.15	0.914	1.46
	Systemic diagnosis in 1985	1.16	0.916	1.47
	Low socioeconomic status	0.790	0.589	1.05
Ketobemidone				
	Periodontitis	3.32	1.40	7.86
	Male	0.541	0.226	1.29
	Smoker	0.631	0.260	1.53
	Systemic diagnosis in 1985	3.48	1.46	8.32
	Low socioeconomic status	1.53	0.619	3.80
Metronidazole				
	Periodontitis	1.46	1.03	2.08
	Male	0.567	0.422	0.762
	Smoker	1.51	1.13	2.03
	Systemic diagnosis in 1985	0.818	0.602	1.11
	Low socioeconomic status	1.01	0.712	1.44

## 4 Discussion

To our knowledge, this is the first study investigating the procurement of medications and specific drug preparations in a 35-year perspective since the diagnosis of periodontitis, compared to the periodontally healthy subjects at baseline. The main finding was that periodontitis patients had purchased certain, but not at all medications, more frequently than we had expected. Hence, the patients did not tend to buy more medications in general, and differences were only seen between the periodontitis and periodontally healthy groups when analyzing the various ATC categories of medicines and the specific preparations within the categories. The results thus only partly confirmed our study hypothesis.

This area of research has been scarcely investigated earlier. Only one prior article was found on the use of systemic medications by periodontitis patients (Wang et al., 2020). Compared to that, our study showed fewer significant results. But it should be pointed out that the study by Wang and collaborators investigated matched subjects while our study was a longitudinal cohort study.

Nevertheless, results similar to those of the study of Wang et al. were found for insulin, oral hypoglycemics in general, ACE inhibitors, calcium channel blockers, lipid-lowering medications, and alpha-2 agonists. However, contrary to Wang et al., we could not establish a connection between periodontitis patients and the use of diuretics, anti-coagulants, bronchodilators, antidepressants, antipsychotic drugs, and anticonvulsants. This indeed can be explained by the differences in the study design and subjects.

As said, hundreds of different systemic medications affect the oral cavity. The main effect is hyposalivation with consequent xerostomia (Tanasiewicz et al., 2016). The salivation-altering medications identified in the present study were angiotensin II receptor blockers, analgesics, anti-infectives, anti-inflammatory medications, alpha-2 agonists, antigout medications, cardiovascular medications like calcium channel blockers, drugs used for diabetes, and those for nicotine dependence, immunosuppressants, and interferons and statins, respectively (Pająk-Łysek et al., 2021; Choo et al., 2022). Hyposalivation is a serious issue because it can increase the risk of diseases of the oral cavity. Poor oral health is also linked to a decrease in the patient’s quality of life (Barbe, 2018).

Xerostomia has been shown to affect 34%–51% of diabetic patients mainly through salivary dysfunction. The linkage between periodontitis and diabetes is well understood because poor glycemic control does worsen periodontal health (Rohani, 2019). Hence, it was no surprise that metformin and the 12 other diabetic medications here encountered were purchased more often by patients with periodontitis.

Merely three systemic individual preparations, namely simvastatin, ketobemidone, and metronidazole showed a positive odds ratio for long-term periodontitis in the present study. No research has been made on a link between opioids or painkillers and periodontitis diagnosis. Similar to our findings, a recent study, however, showed a connection between statins and periodontitis (Kwon et al., 2022). The use of antibiotics as a part of periodontal care varies substantially, necessitating clear guidelines (Feres et al., 2015). In Sweden, antibiotics are rarely used in standard periodontal care. Purchasing metronidazole more often, an antibiotic used mainly for Gram-positive bacteria and protozoa, would thereby indicate that either periodontal disease would increase the risk of infections or infections increase the risk of developing periodontitis. This, however, could not be verified based on the present material.

To account for the impact of long-term systemic medication before 1985 at the onset of this study, patients diagnosed with systemic diseases were identified by using the Swedish national registers for hospital treatment and open care. This was taken into account in the logistic regression analyses and having at least one diagnosis before 1985 was found to significantly increase the risk of purchasing the majority of ATC categories analyzed. Exceptions in this regard were drugs for diseases of the genito-urinary system, sex hormones, anti-infectives for systemic use, drugs for skin diseases, antiparasitic preparations, insecticides and repellents, and, finally, antineoplastic and immunomodulating agents.

A significant difference in the specific medication purchases was seen only in a few drug preparations in this study. This is not in line with current research on the connection between periodontitis and systemic diseases (Liccardo et al., 2019). When compared with patients without periodontitis, there was no significant difference in the purchase of cancer medications, and anti-rheumatic and neurological medication. This finding was unexpected as both different subtypes of cancers, rheumatic and neurological diseases such as Alzheimer’s and depression have been linked to periodontitis (Michaud et al., 2017; Nwizu et al., 2020; Kavarthapu and Gurumoorthy, 2021; Tuominen and Rautava, 2021; Zheng et al., 2021; Asher et al., 2022). Furthermore, the finding is not in line with our earlier research (Söder et al., 2015). However, the present results only represent the sample here used and do not necessarily give the full picture of the whole cohort.

Several covariables have been taken into account in this research. For instance, the purchasing of varenicline, a nicotine-dependence drug, is closely linked with tobacco use. Since a large percentage of the periodontitis patients were smokers, their purchasing varenicline could be expected. Smoking is a well-known risk factor affecting both systemic and periodontal health (Leite et al., 2018). Gender is another important factor as there are differences in oral health between men and women and likewise so when considering socioeconomic status (Boillot et al., 2011; Leng et al., 2015). Interestingly, our odds ratios showed only a few positive results regarding lower socio-economic status and gender, and only partly so concerning tobacco usage.

The strength of the present study is that it offers unique and substantial material with a long follow-up period. The multitude of preparations and a large number of purchases thus made the investigation reliable. This, as well as the relatively big sample size, allowed for conducting the detailed analysis. However, there is room for improvement when planning further studies. The size of the cohort could still be increased if possible. For example, melphalan, vinorelbine, pizotifen, betahistine, and riluzole were only used by one patient in the present material. Thus, there was no way to draw further conclusions in that respect. Furthermore, since the beginning of the study, the diagnostic criteria for periodontitis have been changed several times. Hence, if the most recent diagnostic criteria would have been used the material might look different. This is another weakness of the present study. Nevertheless, we have found a link between medication purchases and periodontitis, especially when looking at the specific preparations and subgroups of medications. Finally, this area has been sparsely studied before and, subsequently, studies with other cohorts are needed.

## 5 Conclusion

We conclude that patients with periodontitis had purchased only a few medication groups more than periodontally healthy subjects when looking at the main drug categories. This finding was contrary to our expectations. On the other hand, periodontitis patients had purchased more than 19 different subgroups of medications. These included diabetes drugs, calcium channel blockers, agents acting on therenin–angiotensin system, statins, and drugs for diseases of the nervous system. Of the specific preparations, only simvastatin, ketobemidone, and metronidazole had been purchased significantly more often by the periodontitis patients. Many of these drugs cause hyposalivation as their side effect which must be taken into account when counseling patients in general.

## Data Availability

The raw data supporting the conclusion of this article will be made available by the authors, without undue reservation.

## References

[B1] Asher S. Stephen R. Mäntylä P. Suominen A. L. Solomon A. (2022). Periodontal health, cognitive decline, and dementia: A systematic review and meta-analysis of longitudinal studies. J. Am. Geriatr. Soc. 70, 2695–2709. 10.1111/jgs.17978 36073186 PMC9826143

[B2] Barbe A. G. (2018). Medication-Induced xerostomia and hyposalivation in the elderly: Culprits, complications, and management. Drugs Aging 35, 877–885. 10.1007/s40266-018-0588-5 30187289

[B3] Boillot A. El Halabi B. Batty G. D. Rangé H. Czernichow S. Bouchard P. (2011). Education as a predictor of chronic periodontitis: A systematic review with meta-analysis population-based studies. PLoS One 6, e21508. 10.1371/journal.pone.0021508 21814546 PMC3140980

[B4] Choo P. J. Taing M. W. Teoh L. (2022). A retrospective study of drugs associated with xerostomia from the Australian Database of Adverse Event Notifications. Int. J. Pharm. Pract. 30, 548–553. 10.1093/ijpp/riac051 36047517

[B5] Feres M. Figueiredo L. C. Soares G. M. Faveri M. 2015. Systemic antibiotics in the treatment of periodontitis. Periodontol. 2000, 67, 131–186. 10.1111/prd.12075 25494600

[B6] Glick A. Sista V. Johnson C. (2020). Oral manifestations of commonly prescribed drugs. Am. Fam. Physician 102, 613–621. 33179891

[B7] Gupta S. Saarikko M. Pfützner A. Räisänen I. T. Sorsa T. (2022). Compromised periodontal status could increase mortality for patients with COVID-19. Lancet Infect. Dis. 22, 314. 10.1016/S1473-3099(22)00065-2 35218744 PMC8865881

[B8] Hansen P. R. Holmstrup P. (2022). Cardiovascular diseases and periodontitis. Adv. Exp. Med. Biol. 1373, 261–280. 10.1007/978-3-030-96881-6_14 35612803

[B9] Humphrey L. L. Fu R. Buckley D. I. Freeman M. Helfand M. (2008). Periodontal disease and coronary heart disease incidence: A systematic review and meta-analysis. J. Gen. Intern Med. 23, 2079–2086. 10.1007/s11606-008-0787-6 18807098 PMC2596495

[B10] Kaur G. Verhamme K. M. Dieleman J. P. Vanrolleghem A. Van Soest E. M. Stricker B. H. (2010). Association between calcium channel blockers and gingival hyperplasia. J. Clin. Periodontol. 37, 625–630. 10.1111/j.1600-051X.2010.01574.x 20642630

[B11] Kavarthapu A. Gurumoorthy K. (2021). Linking chronic periodontitis and oral cancer: A review. Oral Oncol. 121, 105375. 10.1016/j.oraloncology.2021.105375 34140233

[B12] Kwon M. J. Byun S. H. Kim J. H. Kim J. H. Kim S. H. Kim N. Y. (2022). Longitudinal follow-up study of the association between statin use and chronic periodontitis using national health screening cohort of Korean population. Sci. Rep. 12, 5504. 10.1038/s41598-022-09540-y 35365732 PMC8976040

[B13] Leite F. R. M. Nascimento G. G. Scheutz F. López R. (2018). Effect of smoking on periodontitis: A systematic review and meta-regression. Am. J. Prev. Med. 54, 831–841. 10.1016/j.amepre.2018.02.014 29656920

[B14] Leng B. Jin Y. Li G. Chen L. Jin N. (2015). Socioeconomic status and hypertension: A meta-analysis. J. Hypertens. 33, 221–229. 10.1097/HJH.0000000000000428 25479029

[B15] Liccardo D. Cannavo A. Spagnuolo G. Ferrara N. Cittadini A. Rengo C. (2019). Periodontal disease: A risk factor for diabetes and cardiovascular disease. Int. J. Mol. Sci. 20, 1414. 10.3390/ijms20061414 30897827 PMC6470716

[B16] Marouf N. Cai W. Said K. N. Daas H. Diab H. Chinta V. R. (2021). Association between periodontitis and severity of COVID-19 infection: A case-control study. J. Clin. Periodontol. 48, 483–491. 10.1111/jcpe.13435 33527378 PMC8014679

[B17] Meurman J. H. Bascones-Martinez A. (2021). Oral infections and systemic health - more than just links to cardiovascular diseases. Oral Health Prev. Dent. 19, 441–448. 10.3290/j.ohpd.b1993965 34505498 PMC11640876

[B18] Michaud D. S. Fu Z. Shi J. Chung M. (2017). Periodontal disease, tooth loss, and cancer risk. Epidemiol. Rev. 39, 49–58. 10.1093/epirev/mxx006 28449041 PMC5868279

[B19] Miranda-Rius J. Brunet-Llobet L. Lahor-Soler E. Farré M. (2015). Salivary secretory disorders, inducing drugs, and clinical management. Int. J. Med. Sci. 12, 811–824. 10.7150/ijms.12912 26516310 PMC4615242

[B20] Mizutani S. Ekuni D. Tomofuji T. Azuma T. Kataoka K. Yamane M. (2015). Relationship between xerostomia and gingival condition in young adults. J. Periodontal Res. 50, 74–79. 10.1111/jre.12183 24697562

[B21] Närhi T. O. Meurman J. H. Ainamo A. Nevalainen J. M. Schmidt-Kaunisaho K. G. Siukosaari P. (1992). Association between salivary flow rate and the use of systemic medication among 76-81-and 86-year-old inhabitants in Helsinki, Finland. J. Dent. Res. 71, 1875–1880. 10.1177/00220345920710120401 1452886

[B22] Nwizu N. Wactawski-Wende J. Genco R. J. 2020. Periodontal disease and cancer: Epidemiologic studies and possible mechanisms. Periodontol. 2000, 83, 213–233. 10.1111/prd.12329 32385885 PMC7328760

[B23] Orilisi G. Mascitti M. Togni L. Monterubbianesi R. Tosco V. Vitiello F. (2021). Oral manifestations of COVID-19 in hospitalized patients: A systematic review. Int. J. Environ. Res. Public Health 18, 12511. 10.3390/ijerph182312511 34886241 PMC8656958

[B24] Pająk-Łysek E. Polak M. Kopeć G. Podolec M. Desvarieux M. Pająk A. (2021). Associations between pharmacotherapy for cardiovascular diseases and periodontitis. Int. J. Environ. Res. Public Health 18, 770. 10.3390/ijerph18020770 33477530 PMC7831110

[B25] Pussinen P. J. Kopra E. Pietiäinen M. Lehto M. Zaric S. Paju S. 2022. Periodontitis and cardiometabolic disorders: The role of lipopolysaccharide and endotoxemia. Periodontol. 2000, 89, 19–40. 10.1111/prd.12433 35244966 PMC9314839

[B26] Rees T. D. 1998. Drugs and oral disorders. Periodontol. 2000, 18, 21–36. 10.1111/j.1600-0757.1998.tb00136.x 10200710

[B27] Rohani B. (2019). Oral manifestations in patients with diabetes mellitus. World J. Diabetes 10, 485–489. 10.4239/wjd.v10.i9.485 31558983 PMC6748880

[B28] Söder B. Andersson L. C. Meurman J. H. Söder P. (2015). Unique database study linking gingival inflammation and smoking in carcinogenesis. Philos. Trans. R. Soc. Lond B Biol. Sci. 370, 20140041. 10.1098/rstb.2014.0041 25533098 PMC4275910

[B29] Söder P. O. Jin L. J. Söder B. Wikner S. (1994). Periodontal status in an urban adult population in Sweden. Community Dent. Oral Epidemiol. 22, 106–111. 10.1111/j.1600-0528.1994.tb01582.x 8205774

[B30] Tanasiewicz M. Hildebrandt T. Obersztyn I. (2016). Xerostomia of various etiologies: A review of the literature. Adv. Clin. Exp. Med. 25, 199–206. 10.17219/acem/29375 26935515

[B31] Trackman P. C. Kantarci A. (2015). Molecular and clinical aspects of drug-induced gingival overgrowth. J. Dent. Res. 94, 540–546. 10.1177/0022034515571265 25680368 PMC4485217

[B32] Tuominen H. Rautava J. (2021). Oral microbiota and cancer development. Pathobiology 88, 116–126. 10.1159/000510979 33176328

[B33] Wang I. C. Askar H. Ghassib I. Wang C. W. Wang H. L. (2020). Association between periodontitis and systemic medication intake: A case-control study. J. Periodontol. 91, 1245–1255. 10.1002/JPER.19-0593 32077489

[B34] Yuan A. Woo S. B. (2020). Adverse drug events in the oral cavity. Dermatol Clin. 38, 523–533. 10.1016/j.det.2020.05.012 32892860

[B35] Zheng D. X. Kang X. N. Wang Y. X. Huang Y. N. Pang C. F. Chen Y. X. (2021). Periodontal disease and emotional disorders: A meta-analysis. J. Clin. Periodontol. 48, 180–204. 10.1111/jcpe.13395 33103263

