# Supplementary material for: Periodontitis may predict the use of prescription medicines later in life, a database study
Source: Front Pharmacol. 2023 Mar 13;14:1146475. doi: 10.3389/fphar.2023.1146475 (PMC10040546; doi:10.3389/fphar.2023.1146475)
Supplement: Supplementary file 1 [file DataSheet1.docx]

Supplementary Material

**Periodontitis may predict the use of prescription medicines later in life**

**Freja Frankenhaeuser^1*^, Birgitta Söder^2^, Håkan Källmén^3^, Esa R. Korpi^4^, Jukka H. Meurman^1^**

*** Correspondence:** Freja Frankenhaeuser: freja.frankenhaeuser@helsinki.fi

1. **Tables**

**TABLE 1** Basic characteristics of the subjects. The table presents patients that have purchased medicines between the years 2005 - 2017. Data are given as n (%). Only p-values in line with our hypothesis are presented, due to our single sided hypothesis.

|  | Total  (n=1665) | No-periodontitis  (n = 1370) | Periodontitis  (n = 285) | P-value |
| --- | --- | --- | --- | --- |
| Female | 831 | 706 (85.0%) | 125 (15.4%) |  |
| Male | 824 | 664 (80.6%) | 160 (19.4%) | 0.009 |
| Non-smoker | 618 | 907 (86.4%) | 143 (13.6%) |  |
| Smoker | 605 | 463 (76.5%) | 142 (23.5%) | <0.001 |
| No earlier diagnosis | 1070 | 884 (82.6%) | 186 (17.4%) |  |
| Diagnosis in 1985 | 585 | 486 (83.1%) | 99 (16.9%) |  |
| Have not purchased medication | 120 | 90 (75.0%) | 30 (25.0%) |  |
| Have purchased medication(s) | 1535 | 1280 (83.4%) | 255 (16.6%) |  |
| Higher socio-economic status | 1315 | 1097 (83.4%) | 273 (16.6%) |  |
| Lower socio-economic status | 340 | 218 (80.3%) | 67 (19.7%) | 0.087 |

**TABLE 2** Distributions of different medication groups purchased in the ATC categories. Only p-values in line with our hypothesis are presented, due to our single sided hypothesis.

| **Alimentary tract and metabolism** | **Total** | **No periodontitis** | | **Periodontits** | | **p-value** |
| --- | --- | --- | --- | --- | --- | --- |
| Have not purchased | 540 | 441 | (81.7%) | 99 | (18.3%) |  |
| Have purchased | 1115 | 929 | (83.3%) | 186 | (16.7%) |  |
| **Blood and blood forming organs** | |  |  |  |  |  |
| Have not purchased | 889 | 739 | (83.1%) | 150 | (16.9%) |  |
| Have purchased | 766 | 631 | (82.4%) | 135 | (17.6%) | 0.344 |
| **Cardiovascular system** | |  |  |  |  |  |
| Have not purchased | 640 | 539 | (84.2%) | 101 | (15.8%) |  |
| Have purchased | 1015 | 831 | (81.9%) | 184 | (18.1%) | 0.109 |
| **Dermatologicals** |  |  |  |  |  |  |
| Have not purchased | 683 | 566 | (82.9%) | 127 | (18.6%) |  |
| Have purchased | 962 | 804 | (83.6%) | 158 | (16.4%) |  |
| **Genito-urinary system and sex hormones** | |  |  |  |  |  |
| Have not purchased | 835 | 689 | (82.5%) | 146 | (17.5%) |  |
| Have purchased | 820 | 681 | (83.0%) | 139 | (17.0%) |  |
| **Systemic hormonal preparations, excluding sex hormones and insulins** | | | | |  |  |
| Have not purchased | 1078 | 878 | (81.4%) | 200 | (18.6%) |  |
| Have purchased | 557 | 492 | (88.3%) | 85 | (15.3%) |  |
| **Anti-infectives for systemic use** | |  |  |  |  |  |
| Have not purchased | 276 | 228 | (82.6%) | 48 | (17.4%) |  |
| Have purchased | 1379 | 1142 | (82.8%) | 237 | (17.2%) |  |
| **Antineoplastic and immunomodulating agents** | | |  |  |  |  |
| Have not purchased | 1481 | 1231 | (83.1%) | 250 | (16.9%) |  |
| Have purchased | 174 | 139 | (79.9%) | 35 | (20.1%) | 0.143 |
| **Musculo-skeletal system** | |  |  |  |  |  |
| Have not purchased | 502 | 415 | (82.7%) | 87 | (17.3%) |  |
| Have purchased | 1153 | 955 | (82.8%) | 198 | (17.2%) |  |
| **Nervous system** |  |  |  |  |  |  |
| Have not purchased | 458 | 377 | (82.3%) | 81 | (17.7%) |  |
| Have purchased | 1197 | 993 | (83.0%) | 204 | (17.0%) |  |
| **Antiparasitic products, insecticides and repellents** | | |  |  |  |  |
| Have not purchased | 1301 | 1084 | (83.3%) | 217 | (16.7%) |  |
| Have purchased | 354 | 286 | (80.8%) | 68 | (19.2%) | 0.132 |
| **Respiratory system** |  |  |  |  |  |  |
| Have not purchased | 498 | 404 | (81.1%) | 94 | (18.9%) |  |
| Have purchased | 1157 | 966 | (83.5%) | 191 | (16.5%) |  |
| **Sensory organs** |  |  |  |  |  |  |
| Have not purchased | 813 | 668 | (82.2%) | 145 | (17.8%) |  |
| Have purchased | 842 | 702 | (83.4%) | 140 | (16.6%) |  |
| **Various** |  |  |  |  |  |  |
| Have not purchased | 1633 | 1350 | (82.7%) | 283 | (17.3%) |  |
| Have purchased | 22 | 20 | (90.9%) | 2 | (9.1%) |  |

**TABLE 3** Drugs most frequently purchased within the ATC categories. The statistically significant distribution in favor of periodontitis patients are bolded.

| **ATC classification** | **Total** | **Non-periodontitis** | | **Periodontitis** | | **p-value** |
| --- | --- | --- | --- | --- | --- | --- |
| **Alimentary tract and metabolism** | | |  |  |  |  |
| **Drugs used in diabetes** | |  |  |  |  |  |
| Have not purchased | 1499 | 1249 | (83.3%) | 250 | (16.7%) |  |
| Have purchased | 156 | 121 | (77.6%) | 35 | (22.4%) | **0.035** |
| **Cardiovascular system** | | |  |  |  |  |
| **Cardiac therapy** | |  |  |  |  |  |
| Have not purchased | 1444 | 1198 | (83.0%) | 246 | (17.0%) |  |
| Have purchased | 211 | 172 | (81.5%) | 39 | (18.5%) | 0.300 |
| **Beta blocking agents** | |  |  |  |  |  |
| Have not purchased | 1178 | 982 | (83.4%) | 196 | (16.6%) |  |
| Have purchased | 477 | 388 | (81.3%) | 89 | (18.7%) | 0.162 |
| **Calcium channel blockers** | | |  |  |  |  |
| Have not purchased | 1271 | 1066 | (83.9%) | 205 | (16.1%) |  |
| Have purchased | 384 | 304 | (79.2%) | 80 | (20.8%) | **0.016** |
| **Agents acting on the renin–angiotensin system** | | | |  |  |  |
| Have not purchased | 1055 | 887 | (84.1%) | 168 | (15.9%) |  |
| Have purchased | 600 | 483 | (80.5%) | 117 | (19.5%) | **0.032** |
| **Lipid modifying agents** | |  |  |  |  |  |
| Have not purchased | 1139 | 957 | (84.0%) | 182 | (16.0%) |  |
| Have purchased | 519 | 182 | (35.1%) | 103 | (19.8%) | **0.024** |
| **Antineoplastic and immunomodulating agents** | | | |  |  |  |
| **Antineoplastic agents** | |  |  |  |  |  |
| Have not purchased | 1601 | 1328 | (82.9%) | 273 | (17.1%) |  |
| Have purchased | 54 | 42 | (77.8%) | 12 | (22.2%) | 0.161 |
| **Endocrine therapy** | |  |  |  |  |  |
| Have not purchased | 1588 | 1315 | (82.8%) | 273 | (17.2%) |  |
| Have purchased | 67 | 55 | (82.1%) | 12 | (17.9%) | 0.440 |
| **Immunosuppressants** | |  |  |  |  |  |
| Have not purchased | 1601 | 1326 | (82.8%) | 275 | (17.2%) |  |
| Have purchased | 54 | 44 | (81.5%) | 10 | (18.5%) | 0.399 |
| **Musculo-skeletal system** | | |  |  |  |  |
| **Anti-inflammatory and antirheumatic products** | | | |  |  |  |
| Have not purchased | 561 | 467 | (83.2%) | 98 | (17.5%) |  |
| Have purchased | 1094 | 903 | (82.5%) | 191 | (17.5%) | 0.360 |
| **Topical products for joint and muscular pain** | | | |  |  |  |
| Have not purchased | 1484 | 1230 | (82.9%) | 254 | (17.1%) |  |
| Have purchased | 171 | 140 | (81.9%) | 31 | (18.1%) | 0.370 |
| **Muscle relaxants** | |  |  |  |  |  |
| Have not purchased | 1484 | 1231 | (83.0%) | 253 | (17.0%) |  |
| Have purchased | 171 | 139 | (81.3%) | 32 | (18.7%) | 0.293 |
| **Antigout preparations** | |  |  |  |  |  |
| Have not purchased | 1587 | 1316 | (82.9%) | 271 | (17.1%) |  |
| Have purchased | 68 | 54 | (79.4%) | 14 | (20.6%) | 0.227 |
| **Nervous system** | |  |  |  |  |  |
| **Analgesics** | |  |  |  |  |  |
| Have not purchased | 643 | 538 | (83.7%) | 105 | (16.3%) |  |
| Have purchased | 1012 | 832 | (82.2%) | 180 | (17.8%) | 0.222 |
| **Antiepileptics** | |  |  |  |  |  |
| Have not purchased | 1526 | 1268 | (83.1%) | 258 | (16.9%) |  |
| Have purchased | 129 | 102 | (79.1%) | 27 | (20.9%) | 0.123 |
| **Anti-parkinson drugs** | |  |  |  |  |  |
| Have not purchased | 1607 | 1331 | (82.8%) | 276 | (17.2%) |  |
| Have purchased | 48 | 39 | (81.3%) | 9 | (18.8%) | 0.388 |
| **Psycholeptics** | |  |  |  |  |  |
| Have not purchased | 974 | 807 | (82.9%) | 167 | (17.1%) |  |
| Have purchased | 681 | 563 | (82.7%) | 118 | (17.3%) | 0.462 |
| **Other nervous system drugs** | | |  |  |  |  |
| Have not purchased | 1548 | 1293 | (83.5%) | 255 | (16.5%) |  |
| Have purchased | 107 | 77 | (72.0%) | 30 | (28.0%) | **0.001** |
| **Antiparasitic products, insecticides and repellents** | | | |  |  |  |
| **Antiprotozoals** | |  |  |  |  |  |
| Have not purchased | 1312 | 1094 | (83.4%) | 218 | (16.6%) |  |
| Have purchased | 343 | 276 | (80.5%) | 67 | (19.5%) | 0.102 |

**TABLE 4** Linear regressions and odds ratios (OR) with confidence intervals (CI) of drug categories associated with having periodontitis in 1985.

|  | Cofactors | OR | 95% CI for OR | |
| --- | --- | --- | --- | --- |
| Nervous system | |  | Lower | Upper |
|  | Periodontitis | .957 | .714 | 1.28 |
|  | Male | .553 | .442 | .692 |
|  | Smoker | **1.49** | **1.17** | **1.89** |
|  | Prior diagnosis to 1985 | **1.54** | **1.21** | **1.96** |
|  | Lower socioeconomic | .634 | .487 | .825 |
| Alimentary tract and metabolism | | |  |  |
|  | Periodontitis | .932 | .707 | 1.22 |
|  | Male | .612 | .495 | .756 |
|  | Smoker | 1.06 | .850 | 1.32 |
|  | Prior diagnosis to 1985 | **1.61** | **1.28** | **2.02** |
|  | Lower socioeconomic | .653 | .507 | .840 |
| Cardiovascular system | |  |  |  |
|  | Periodontitis | 1.16 | .888 | 1.52 |
|  | Male | .989 | .808 | 1.20 |
|  | Smoker | 1.19 | .969 | 1.47 |
|  | Prior diagnosis to 1985 | **1.44** | **1.16** | **1.78** |
|  | Lower socioeconomic | .796 | .622 | 1.01 |
| Antineoplastic and immunomodulating agents | | | |  |
|  | Periodontitis | 1.27 | .852 | 1.90 |
|  | Male | .633 | .457 | .877 |
|  | Smoker | 1.12 | .809 | 1.55 |
|  | Prior diagnosis to 1985 | 1.25 | .903 | 1.73 |
|  | Lower socioeconomic | .814 | .538 | 1.22 |

**TABLE 5** Linear regressions and odds ratios (OR) with confidence intervals (CI) of individual

medications associated with having periodontitis in 1985.

|  | Cofactors | OR | 95% CI for OR | |
| --- | --- | --- | --- | --- |
| Simvastatin | |  |  |  |
|  | Periodontitis | **1.39** | **1.04** | **1.86** |
|  | Male | 1.14 | .909 | 1.44 |
|  | Smoker | 1.15 | .914 | 1.46 |
|  | Systemic diagnosis in 1985 | 1.16 | .916 | 1.47 |
|  | Low socioeconomic status | .790 | .589 | 1.05 |
| Ketobemidone | |  |  |  |
|  | Periodontitis | **3.32** | **1.40** | **7.86** |
|  | Male | .541 | .226 | 1.29 |
|  | Smoker | .631 | .260 | 1.53 |
|  | Systemic diagnosis in 1985 | **3.48** | **1.46** | **8.32** |
|  | Low socioeconomic status | 1.53 | .619 | 3.80 |
| Metronidazole | |  |  |  |
|  | Periodontitis | **1.46** | **1.03** | **2.08** |
|  | Male | **.567** | **.422** | **.762** |
|  | Smoker | **1.51** | **1.13** | **2.03** |
|  | Systemic diagnosis in 1985 | .818 | .602 | 1.11 |
|  | Low socioeconomic status | 1.01 | .712 | 1.44 |

1. **Figures**


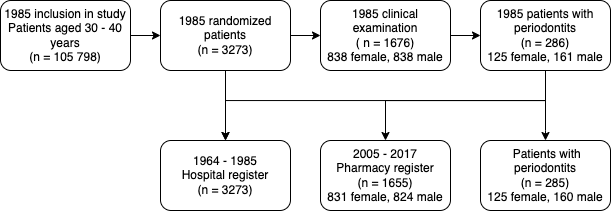


Figure 1. Flow chart of cohort and registers available. In the present study, we mainly focused on the pharmacy register.
